# The optimized PBAE-loaded pro-neurogenic IFs nanoparticles reprogram astrocytes within the glial scar into diverse neuronal subtypes

**DOI:** 10.1186/s13036-026-00755-5

**Published:** 2026-08-08

**Authors:** Zijian Liu, Jin Chen, Xiaoyu Ma, Dandan Zhang, Wenxin Su, Quanrui Ma, Hao Yang

**Affiliations:** 1 Department of Human Anatomy and Histoembryology, School of Basic Medical Sciendes, Ningxia Medical University, Yinchuan, 750004 China; 2 Translational Medicine Center, Hong Hui Hospital, Xi’an Jiaotong University, Xi’an, 710054 China

**Keywords:** PBAE, ASCL1/NGN2, Astrocytes, Glial scar, Reprogramming, Neurons

## Abstract

Glial scar formation and neuronal loss following spinal cord injury (SCI) are major impediments to neural regeneration. Therefore, remodeling the inhibitory scar obstacles and replenishing lost neurons are paramount for neural repair and functional recovery. However, current prevailing therapeutic approaches faced formidable challenges in concurrently achieving effective neuronal replenishment and glial scar (GS) remodeling. Herein, we described an innovative “remodeling-regeneration integrated” non-viral therapeutic strategy designed to directly reprogram reactive astrocytes within GS into functional neurons of distinct subtypes in vitro. This reprogramming is achieved by utilizing poly(β-amino ester) (PBAE) nanoparticles for the efficient co-delivery of the proneural transcription factors ASCL1 and NGN2 (TFs-A/N) into glial scar cells. For the delivery of two genes, the PBAE polymer was synthesized under an optimized Michael addition reaction condition. Our results demonstrated that this polymer possesses low toxicity, favorable biodegradability, and high molecular weight, allowing efficient encapsulation of substantial amounts of TFs-A/N-encoding plasmids into stable nanoparticles. Importantly, these optimized nanoparticles exhibited high gene delivery efficiency. Of particular significance, PBAE-TFs-A/N nanoparticles not only effectively remodeled GS architecture but also induced the generation of diverse neuronal subtypes, including cholinergic, glutamatergic, GABAergic, and dopaminergic neurons, as evidenced by subtype-specific biochemical marker expression. Furthermore, the converted cells acquired neuronal functionality, demonstrated by expression of synaptic markers (SYN and PSD95), action potential firing, and neuron-specific sodium and potassium currents. These findings provide compelling evidence for a safe and effective non-viral reprogramming methodology that simultaneously promotes multi-subtype neuron generation and GS remodeling. Collectively, the study positions the approach as a promising avenue for novel insights into CNS injury repair and clinical translational applications.

**Supplementary Information:**

The online version contains supplementary material available at https://doi.org/10.1186/s13036-026-00755-5.

## Introduction

 Spinal cord injury (SCI), caused by traumatic (e.g., falls, accidents) or non-traumatic (e.g., tumors, infections) insults, is characterized by severe disruption of spinal cord structure and function, frequently leads to loss of sensory and motor functions, is accompanied by autonomic disorders, and physiological dysfunction [1]. The global prevalence of SCI is steadily rising, with traumatic SCI (TSCI) being the most common type [2]. China currently has approximately 759,302 individuals living with TSCI, with 66,374 new cases annually [3], largely attributed to traffic accidents, falls, and blunt force trauma [4]. SCI not only causes profound physical and psychological distress for patients but also imposes a substantial socioeconomic burden. Given the complex pathophysiology of SCI progression, current interventions (e.g., surgery, glucocorticoids) can only partially preserve residual neural function but fail to reverse neuronal loss at the injury site, which is the core pathological deficit of SCI. Therefore, developing innovative therapies to achieve effective neural repair has become an urgent and unmet clinical need in this field.

The pathological cascade following SCI is highly complex and dynamic. The primary injury triggers a series of deleterious events, including microglial activation, inflammatory responses, oxidative stress, and release of proteolytic enzymes, which typically exacerbate lesion progression and lead to lesion expansion and extensive neuronal apoptosis [5, 6]. Among these, reactive astrocytic hypertrophy and proliferation around the injury site collectively contribute to the formation of a glial scar (GS). Although the GS can physically isolate the injury and limit its further spread during the early stage of SCI, its persistent physical and chemical barrier ultimately impedes axonal regeneration and neural circuit reconstruction [7]. Notably, astrocytes, as the primary cellular components of the GS, have recently been regarded as a promising endogenous cell source for neural regeneration due to their widespread distribution and plasticity, which enables them to differentiate into multipotent neural stem cells (NSCs) or even specific neuronal subtypes through forced expression of defined transcription factors (TFs) [2, 8–10] or chemical stimulation [11–13]. More strikingly, accumulating studies reveal that targeted expression of specific transcription factors (e.g., NeuroD1, NG2, and LMX1A) can directly reprogram astrocytes into functional neurons of certain subtypes [14–17], providing a novel research direction for in situ neural repair based on astrocyte reprogramming. Thus, astrocytes are likely to be a more promising endogenous source of cells for the treatment of CNS injuries. Among various neurogenic TFs, the basic helix-loop-helix (bHLH) transcription factor family members ASCL1 and NGN2 demonstrate strong synergistic reprogramming effects [18]. As demonstrated by Chanda et al. [19], overexpression of ASCL1 alone can largely reprogram somatic cells into the neuronal lineage. However, these induced neurons exhibit relatively lower levels of maturity compared to those generated using ASCL1 in combination with other factors such as Neurogenin 2 (NGN2), another member of the bHLH family. Notably, their combination application not only efficiently induces the conversion of astrocytes into neuronal lineages but also significantly enhances the maturity, cell fate determinant, and functional integration potential of the newly generated neurons, exhibiting better overall effects than single-factor intervention [20]. Nevertheless, safely, efficiently, and controllably delivering the ASCL1 and NGN2 genes to target cells within the CNS remains a key technical bottleneck. Although viral vectors offer high transfection efficiency [21], their inherent immunogenicity, potential tumorigenicity, and limited genome packaging capacity [22, 23] restrict their prospects for clinical translation. Therefore, developing safe and efficient non-viral delivery systems is essential.

Considering the requirements of epigenetic regulation, an ideal gene vector should exhibit low cytotoxicity, biodegradable in vivo, protect loaded genes from extracellular degradation, and enable efficient gene delivery and release. Poly(beta-amino ester) (PBAE) is a cationic polymer synthesized with acrylic esters and amine monomers via Michael addition reactions. The polymer possesses several advantageous properties: hydrolytic biodegradability, low toxicity, straightforward synthesis, and ease of structural modification [24]. The tertiary amine groups on its molecular chains enable electrostatic binding to DNA, forming nanoscale complexes. These self-assembled nanoparticle complexes carry positive surface charges, thereby facilitating cellular uptake through adsorption and endocytosis [25]. Moreover, PBAE’s pH-responsive amino groups enable charge reversibility, further enhancing delivery efficiency [26]. In the present study, we focused on this highly promising non-viral gene carrier, PBAE. By optimizing the synthesis process, we successfully developed a PBAE nanoparticle delivery system loaded with ASCL1 and NGN2 plasmids. Leveraging its excellent biocompatibility, biodegradability, pH responsiveness, and high gene-loading capacity, this system aims to achieve the following core objectives: (1) efficiently mediating intracellular delivery and expression of ASCL1 and NGN2 within the glial scar microenvironment, thereby directly driving the in situ conversion of astrocytes into functional neurons; and (2) not only effectively replenishing lost neurons but also remodeling the inhibitory microenvironment and barrier properties of the GS. This dual approach lays a foundation for axonal regeneration and neural circuit reconstruction, ultimately synergistically enhancing spinal cord functional recovery from two dimensions: “cell replenishment” and “microenvironment remodeling”. This study will provide an innovative and translationally promising nanotherapeutic strategy for the regenerative repair of SCI, while also establishing a theoretical and experimental foundation for future clinical implementation.

## Materials and methods

### Main reagents

The following chemicals were purchased for the experiment: 1,4-Butanediol diacrylate (B4), 4-amino-1-butanol (S4), 3-Aminopropyl-4-methylpiperazine (E7), and anhydrous tetrahydrofuran (THF) were obtained from Aladdin Company. Dimethyl sulfoxide (DMSO) and TAE (50x) were purchased from Solarbio Company; agarose powder was supplied by Meilunbio Company; the DL 15,000 DNA marker was procured from VaZyme. Diethyl ether was acquired from Honghua Chemical Glass Co., Ltd. High glucose (DMEM) medium, fetal bovine serum (FBS), Neurobasal cell culture medium, and B-27™ supplement (50X) serum-free were purchased from Gibco; Poly-L-Lysine Hydrobromide was obtained from NEST Biotechnology. Primary and secondary antibodies were sourced from different commercial suppliers: Mouse anti-Glial Fibrillary Acidic Protein (GFAP), rabbit anti-Neurocan, rabbit anti-Choline Acetyltransferase (ChAT), mouse anti-β-3 Tubulin (Tuj-1), rabbit anti-doublecortin (DCX), and rabbit anti-microtubule-associated protein 2 (Map-2), together with Alexa Fluor^®^ 488/594/647-conjugated goat anti-mouse and goat anti-rabbit IgG secondary antibodies were purchased from Invitrogen. Rabbit anti-Vimentin antibody was obtained from Cell Signaling Technology; mouse anti-β-actin antibody was sourced from Zhongshan Jinqiao Company. Rabbit anti-tyrosine hydroxylase (TH), rabbit anti-vesicular glutamate transporter 2 (vGluT2), Synaptophysin (Syn), rabbit anti-postsynaptic density protein 95 (PSD95), and rabbit anti-calcitonin gene-related peptide (CGRP), as well as goat anti-rabbit IgG (Alexa Fluor^®^ 647) and goat anti-mouse IgG (Alexa Fluor^®^ 405), were purchased from Abcam.

### Synthesis and identification of PBAE nanoparticles

The PBAE polymer was synthesized via a two-step polymerization reaction using three distinct monomers: B4 (ester monomer), S4 (amine monomer), and E7 (capping agent). Briefly, B4 and S4 were meticulously mixed at a 1.1:1 molar ratio and stirred thoroughly at 110 °C, 800 rpm, under light-shielded conditions for 4.5 h to yield intermediate i-PBAE. Concomitantly, i-PBAE was dissolved in anhydrous tetrahydrofuran (THF), capped with an equimolar amount of E7, and stirred at room temperature for 1–2 h. Following completion of the reaction, the product was precipitated in pre-chilled anhydrous diethyl ether and washed with diethyl ether three times to remove impurities. The final solid was vacuum-dried for 48 h and stored at -20 °C for subsequent use. For experiment applications, the PBAE polymer was dissolved in DMSO to prepare a 100 mg/mL stock solution and then stored at -20 °C.

The chemical structure of the synthesized product was characterized by ¹H nuclear magnetic resonance (¹H NMR) and Fourier transform infrared spectroscopy (FT-IR). The molecular weight and distribution were determined by gel permeation chromatography (GPC). The cytotoxicity of PBAE nanoparticles was evaluated using the CCK-8 cell viability assay as previously described.

### Construction of plasmids and characterization of PBAE-plasmid nanoparticle complexes

To construct ASCL1 and NGN2 overexpression plasmids, a mammalian expression vector was employed as the backbone, into which the full-length rat GFAP (rGFAP) promoter sequence was inserted into the vector to drive astrocyte-specific expression. Subsequently, rat ASCL1 and NGN2 cDNAs were amplified using high-fidelity DNA polymerase and cloned downstream of the rGFAP promoter, generating two independent plasmids: pcDNA-rGFAP-ASCL1 and pcDNA-rGFAP-NGN2. To facilitate intuitive identification of successfully transfected cells and prevent transcriptional truncation of the target gene, eGFP and mCherry reporters were separately fused downstream of ASCL1 and NGN2, respectively, yielding the final constructs: pcDNA-rGFAP-ASCL1-CMV-eGFP-r and pcDNA-rGFAP-NGN2-CMV-mCherry-r. All plasmids were validated by DNA sequencing, confirming complete sequence integrity and full consistency with the design specifications, with no mutations detected. Following verification of the constructed plasmids by restriction enzyme analysis and DNA sequencing, the plasmids were subsequently transformed into 5-alpha E. coli competent cells. After 24 h incubation on selective plates, five individual colonies were randomly picked for PCR screening. Plasmids extracted from positive clones were used for transfection into the C6 cell line.

PBAE-plasmid nanoparticle complexes were prepared via electrostatic self-assembly. In brief, PBAE was dissolved in sterile ultrapure water (pH 7.2) and vortexed to a homogenous complex solution. The plasmid was then added at a predetermined mass ratio, and the mixture was vortexed for 10 s and incubated at room temperature for 15 min to allow for self-assembly of the nanoparticle complex. To determine the optimal plasmid-to-PBAE mass ratio for efficient complexation, agarose gel electrophoresis was performed using the following plasmid-to-PBAE ratios: 1:0 (plasmid alone), 1:10, 1:20, 1:30, 1:40, 1:50, 1:60, and 1:70. This approach served to evaluate binding efficiency and identify the optimal ratio for complex formation, which was then consistently applied in all subsequent experiments. The morphology, particle size, and uniformity of the prepared nanoparticles were characterized by transmission electron microscopy (TEM). The overall morphology, particle sizes and uniformity of the prepared nanoparticles were characterized and observed via transmission electron microscopy (TEM).

### Astrocyte culture and scratch injury model preparation

Primary spinal cord astrocytes were isolated from postnatal day 1–2 Sprague-Dawley (SD) rats. Rat pups were deeply anesthetized by an ice bath. Under sterile conditions, spinal cord tissue from the cervical to lumbar segments was dissected, with the central canal removed. The spinal cord tissue was immediately placed in prechilled Ca^2+^- and Mg^2+^-free Dulbecco’s Hank’s Balanced Salt Solution (D-Hank’s solution). After removal of the epineurium and blood vessels, the spinal cord tissue was minced and digested with 0.25% EDTA-trypsin solution at 37 °C for 20 min. The digestion reaction was terminated by adding high-glucose DMEM containing 10% FBS. Single-cell suspensions were obtained by gentle mechanical trituration, filtration, and centrifugation at 1000 rpm for 5 min. Finally, the cell pellet was resuspended and seeded on poly-L-lysine-coated culture flasks at a density of 2 × 10^5^ cells/cm^2^ in DMEM containing 15% FBS, 100 U/ml penicillin, and 100 µg/ml streptomycin, and maintained at 37 °C in a 5% CO₂ incubator. Half of the medium was refreshed every three days. When cell confluence exceeded 90%, astrocytes were purified by shaking at 200–280 rpm for 15–20 h at 37 °C. Subsequently, the purified cultures were maintained for the following experiments.

To mimic the glial scar microenvironment following SCI, an in vitro astrocyte scratch injury model was established. When purified astrocytes reached approximately 90% confluence, a standardized grid scratch wound was created at 0.5 cm intervals using a sterile 10 µL pipette tip, following the experimental protocol previously reported by Yang et al. [27]. The cells were returned to the incubator for further culture.

### In vitro transfection of PBAE-loading NGN2/NSCL1 nanoparticle

Prior to the transfection, astrocytes were synchronized with serum starvation in FBS-free DMEM medium for 2 h to enhance transfection efficiency. The prepared PBAE-loaded ASCL1/NGN2 plasmid nanoparticle complex was diluted in FBS-free DMEM medium and gently added into the starved astrocyte cultures. The cells were incubated with the nanoparticle complex for 6 h at 37 °C in a 5% CO₂ incubator. Subsequently, the medium containing nanoparticles was replaced with fresh complete DMEM medium containing 10% FBS, and the cells were cultured under standard conditions for an additional 48 h. The culture medium was then refreshed with neuronal maintenance medium (Neurobasal medium supplemented with 1% B27, 1% Glutamax, and 1% penicillin-streptomycin). Cells were maintained in this medium until the designated experimental timepoints for subsequent functional detection and analysis.

### Immunofluorescence

Cells cultured on coverslips were fixed with 4% paraformaldehyde at room temperature for 10 min, permeabilized with 0.1% Triton X-100 in PBS for 10 min, and blocked with 2% bovine serum albumin (BSA) and 5% corresponding host serum in PBS for 1 h at room temperature. After three washes with PBS (5 min each), the samples were incubated overnight at 4 °C with primary antibodies diluted in PBS containing 1% BSA. The primary antibodies used included mouse anti-GFAP, rabbit anti-GFAP, rabbit anti-Neurocan, rabbit anti-NG2, rabbit anti-ChAT, mouse anti-Tuj-1, rabbit anti-Vimentin, rabbit anti-DCX, rabbit anti-Map-2, rabbit anti-Nestin, rabbit anti-TH, rabbit anti-vGluT2, rabbit anti-NMDAR1, rabbit anti-ParV, rabbit anti-SYN, rabbit anti-PSD95, and rabbit anti-CGRP. After primary antibody incubation, samples were extensively washed three times with PBS and then incubated with the corresponding Alexa Fluor 405/488/594/647-conjugated secondary antibodies (anti-mouse IgG, anti-rabbit IgG, and anti-goat IgG) diluted to 1:400 in PBS for 1 h at room temperature in the dark. Following final thorough washes with PBS, coverslips were mounted on glass slides using DAPI-containing anti-fade mounting medium. All images were captured using a fluorescence microscope with consistent parameters to ensure uniformity across samples.

### Western blot

Cells from different treatments were rinsed with ice-cold PBS and fully lysed on ice for 30 min in RIPA lysis buffer supplemented with PMSF and protease inhibitors. During lysis, the cells were scraped continuously to ensure complete lysis. The lysates were collected and homogenized using a tissue homogenizer (three cycles of 60 s each at 70 Hz), followed by centrifugation at 12,000 × g for 10 min at 4 °C. The clarified supernatants were collected, and protein concentrations were determined using the bicinchoninic acid (BCA) assay. Western blot analysis was performed strictly in accordance with the previously described [28]. Briefly, equal amounts of protein were separated by 10% SDS-PAGE electrophoresis and transferred onto a polyvinylidene fluoride (PVDF) membrane. After rapid blocking for 15 min, membranes were incubated overnight at 4 °C with primary antibodies diluted in TBST. The primary antibodies used in this study included mouse anti-β-actin, mouse anti-GAPDH, mouse anti-GFAP, rabbit anti-Neurocan, rabbit anti-NG2, rabbit anti-ChAT, mouse anti-Tuj-1, rabbit anti-Vimentin, rabbit anti-DCX, rabbit anti-Map-2, rabbit anti-TH, rabbit anti-vGluT2, rabbit anti-SYN, rabbit anti-PSD95, and rabbit anti-CGRP. The following day, the membranes were washed three times with TBST and incubated with HRP-conjugated secondary antibodies (1:10,000 in TBST) for 2 h at room temperature with gentle shaking. Finally, immunoreactive bands were visualized using enhanced chemiluminescence (ECL) reagent and imaged with a gel imaging system.

### Electrophysiology

The transdifferentiated cells were transferred to artificial cerebrospinal fluid (ACSF) and subjected to whole-cell patch-clamp electrophysiological recordings at designated time points post-transfection. All experiments were conducted at room temperature (22–24 °C) according to our previous report [29, 30]. The standard ACSF contained the following (in mM): 140 NaCl, 5 KCl, 2 CaCl₂, 1 MgCl₂, 10 HEPES, and 10 glucose (pH adjusted to 7.4 with NaOH, osmolarity ~ 300 mOsm/kg). Patch pipettes were pulled to a resistance of 3–5 MΩ and filled with the intracellular solution containing (in mM): 130 K-gluconate, 5 KCl, 2 MgATP, 0.3 NaGTP, 1 EGTA, and 10 HEPES (pH adjusted to 7.2 with KOH, osmolarity ~ 290 mOsm/kg). Action potentials (APs) were elicited in current-clamp mode by injecting a series of depolarizing current steps (300 ms duration, 10–100 pA amplitude, 10 pA increments) to assess neuronal excitability. Whole-cell sodium and potassium currents were elicited and recorded in voltage-clamp mode from a holding potential of − 70 mV, with voltage steps applied from − 100 mV to + 50 mV (300 ms duration, 10 mV increments). Data were acquired using an Axopatch 200B amplifier (Molecular Devices) and analyzed with pClamp 10.7 software (Molecular Devices). Only cells with a resting membrane potential more negative than − 50 mV were included in the final analysis.

### Statistical analysis

Data are presented as mean ± standard deviation (SD) from at least three independent experiments. For comparisons between two groups, statistical significance was assessed using an unpaired Student’s t-test. For multiple group comparisons, one-way analysis of variance (ANOVA) followed by Tukey’s post hoc test was employed. If data did not meet the assumptions of normality (as determined by normality tests), the non-parametric Kruskal–Wallis test was adopted. A P value of less than 0.05 was considered statistically significant. Significance levels are defined as follows: *P < 0.05, **P < 0.01, ***P < 0.001, and ****P < 0.0001.

## Result

### Synthesis, characterization of PBAE, and identification of nanoparticles loaded with ASCL1/NGN2 plasmids

**Fig. 1:**
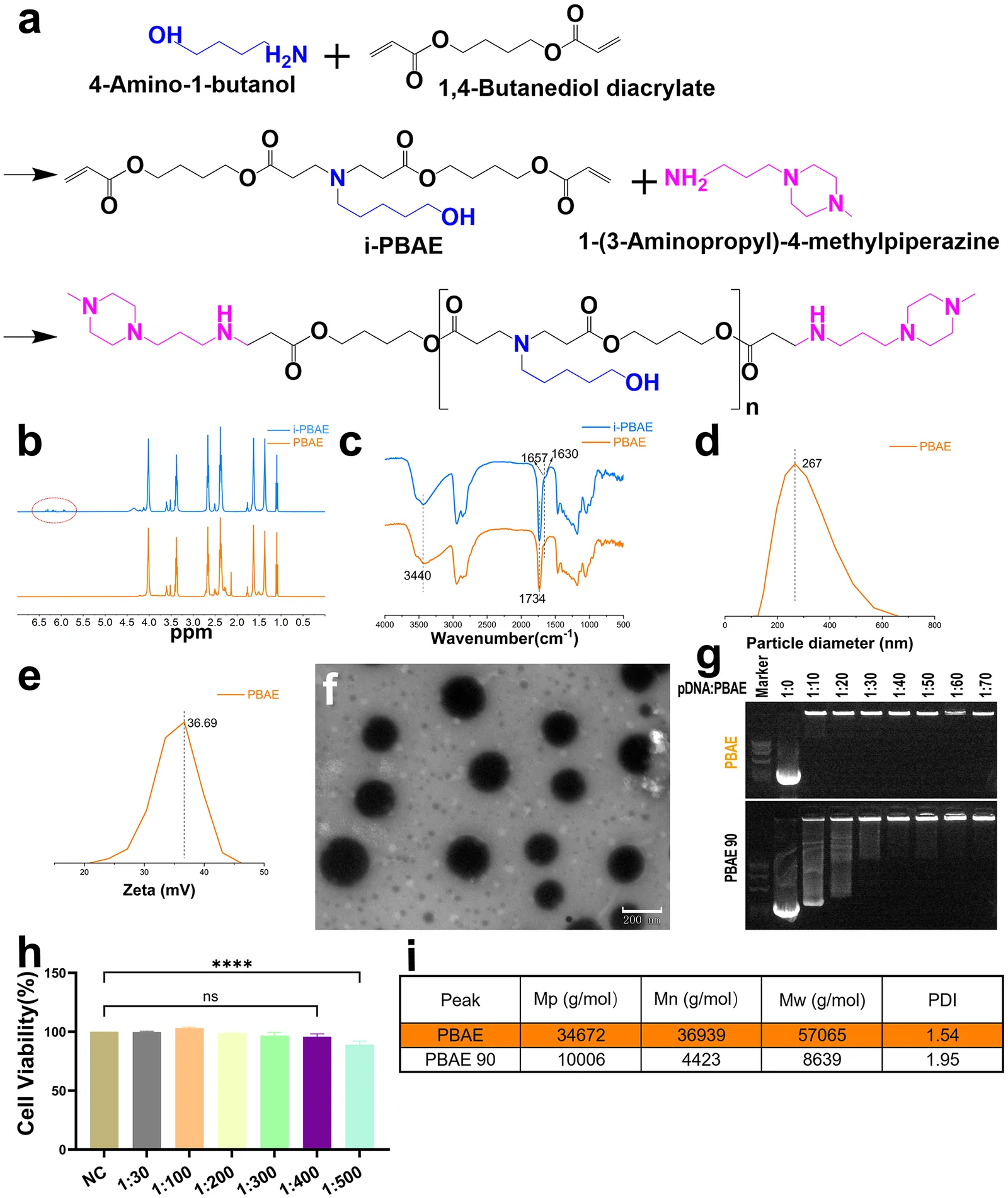
Synthesis and Characterization of PBAE and PBAE-pcDNA Complex Nanoparticles. (**a**) PBAE synthesis scheme. (**b**) ¹H NMR spectra of i-PBAE (intermediate PBAE) and PBAE. (**c**) FTIR spectra of i-PBAE and PBAE. (**d**) Size distribution of PBAE-pcDNA nanoparticles at a pDNA/PBAE mass ratio of 1:20. (**e**) Zeta potential of PBAE-pcDNA nanoparticles at a pDNA/PBAE mass ratio of 1:20. (**f**) TEM image of PBAE-pcDNA nanoparticles at a pDNA/PBAE mass ratio of 1:20 (scale bar = 200 nm). (**g**) Agarose gel electrophoresis retardation assay of pcDNA complexed with PBAE or PBAE 90 at different mass ratios. (**h**) CCK-8 cell viability assay of PBAE-pcDNA nanoparticles at different pcDNA/PBAE mass ratios (*n* = 3 per group, ^****^P < 0.0001 *vs. Control*). (**i**) GPC profiles of PBAE and PBAE

PBAE was synthesized via Michael addition reactions between amine monomers and ester monomers (Fig. 1a). Conventional methods reported in literature [31, 32] require continuous reaction heating at above 90 °C for more than 24 h, highlighting the critical role of hightemperature in driving polymerization reactions. However, extended high-temperature reactions can trigger undesirable side reactions, including thermal polymerization of double bonds or amidation. To mitigate these limitations, we modified the original protocol by increasing the temperature and shortening the reaction time to 4.5 h. To validate the polymer structure and evaluate the modification efficacy, ¹H NMR analysis was performed on three samples: PBAE is synthesized via the conventional method at 90 °C (PBAE 90), the intermediate is isobutene-PBAE (i-PBAE), and the final product is obtained from the aforementioned modified high-temperature short-duration method. The spectra confirmed characteristic chemical shifts for all three polymers, consistent with reported structures [33, 34]. Notably, the i-PBAE spectrum exhibited hydrogen signals in the range of 5.80–6.50 ppm, which were attributable to allyl end groups of the intermediate. In the spectrum of the fully reacted PBAE (Fig. 1b), these signals completely disappeared, substantiating a successful reaction between the i-PBAE intermediate and the E7 acrylate terminator. Furthermore, the FTIR spectrum of i-PBAE showed characteristic absorption peaks corresponding to C = C double-bond stretching vibrations that appeared at 1630–1657 cm^−1^, which were absent in the final PBAE product (Fig. 1b). This aligns with the disappearance of allyl hydrogen signals in ¹H NMR analysis, providing additional evidence for successful PBAE synthesis. A broad absorption peak at 3200–3500 cm^−1^ in the FTIR spectrum indicated the presence of residual -OH groups on the polymer backbone (Fig. 1c).

To further test our hypothesis, we first constructed and sequenced ASCL1 and NGN2 expression plasmids. As shown in Supplementary Fig. 1, the sequences of both ASCL1 and NGN2 plasmids were confirmed to be correct by alignment with expected reference profiles (Figure S1-a and b). Additionally, restriction endonuclease digestion and agarose gel electrophoresis verified the accurate construction of the ASCL1 and NGN2 expression plasmids (Figure S1-c). Furthermore, a fluorescence microscope revealed ASCL1 and NGN2 expression in C6 cells at 1 day post-transfection (Figure S1-d and e). The surface charge and particle size of nanoparticles (NPs) substantially influence cellular uptake efficiency, prompting us to conduct a comprehensive characterization. The NPs showed an average hydrodynamic diameter of 258.7 nm, a zeta potential of + 32.5 mV, and a polydispersity index (PDI) of 0.116, indicating uniform particle size distribution and a stable colloidal system (Fig. 1d and e). Concurrently, transmission electron microscopy (TEM) confirmed spherical or ellipsoidal morphology with excellent dispersion status and uniform particle size distribution (Fig. 1f). Multiple independent measurements of particle size and charge yielded consistent results, fully demonstrating excellent reproducibility and stability of the optimized PBAE synthesis protocol. Cytotoxicity assessment revealed significant cellular toxicity only at pcDNA/PBAE ratios as high as 1:500 (Fig. 1h), with statistically significant differences.

### Identification and characterization of astrocytes and scratch-injury scar models

**Fig. 2:**
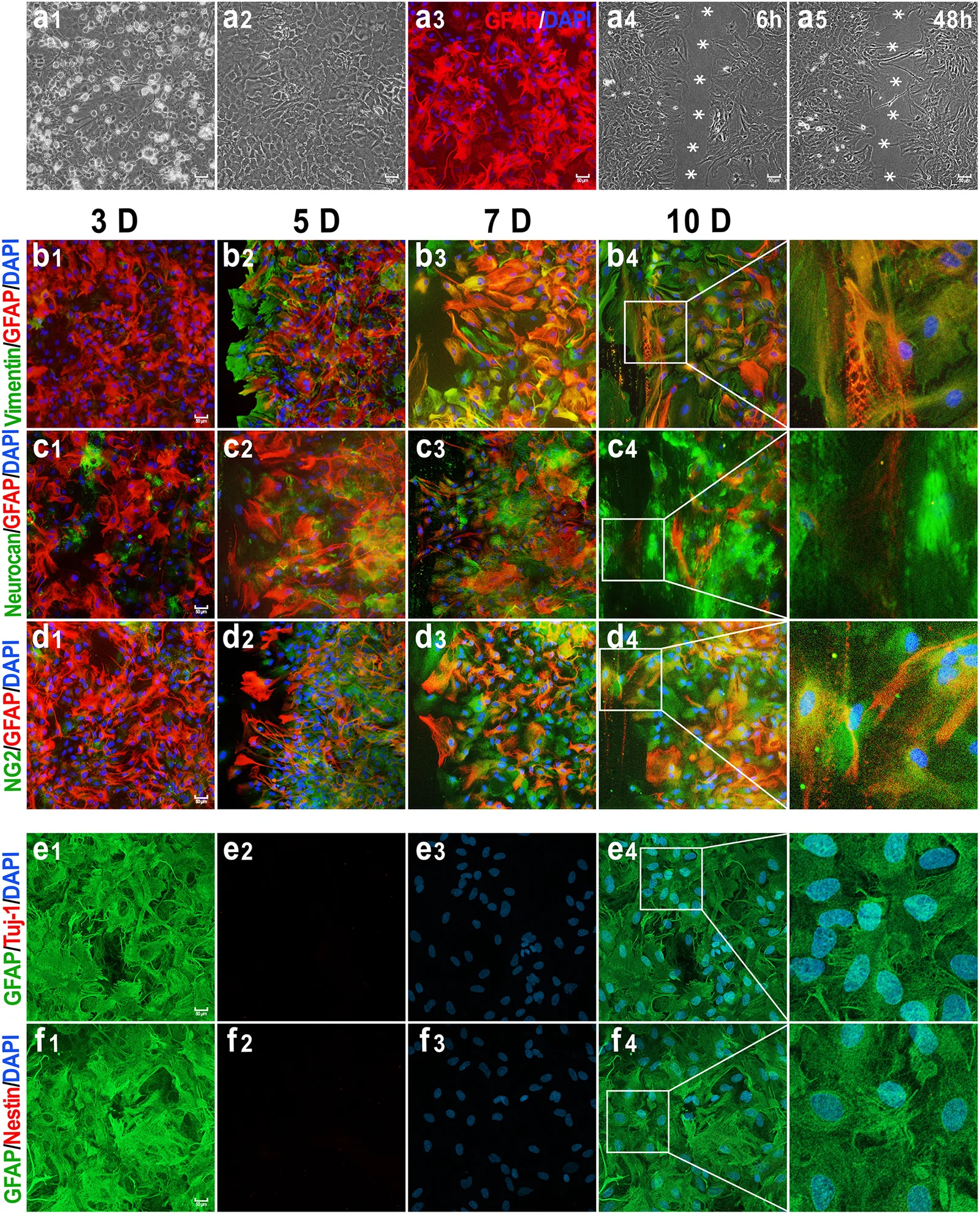
In vitro culture, purification, and glial scar model establishment of astrocytes. (**a1**-**a2**) Phase-contrast microscopy of in vitro cultured astrocytes (**a1**) and their morphology following purification and subculture. (**a3**) Identification of cell purity via immunofluorescence staining for GFAP. (**a4**-**a5**) Phase-contrast images showing astrocyte migration and morphological changes following mechanical scratch induction, depicting the injury model at 6 h (**a4**) and 48 h (**a5**), respectively. (**b1**-**d4**) Immunofluorescence co-staining for GFAP with Vimentin, Neurocan, and NG2, respectively, illustrating the expression of the diffuse deposition of this CSPG component around the scratch area post-injury (scale bar = 50 μm). (**e1**-**f4**) Immunofluorescence co-staining of GFAP with Tuj1 and Nestin (scale bar = 50 μm)

To investigate whether PBAE-loaded-A/N TF nanoparticles (PBAE-A/N NPs) effectively potentiate astrocyte-to-neuron reprogramming, we first isolated, cultured, and identified primary astrocytes from rat spinal cords. After 10 days in vitro, phase-contrast microscopy revealed that most cells exhibited a flat, polygonal morphology and had formed a confluent monolayer, with a subset of cells scattered across the monolayer exhibiting distinct features, including small soma, strong refractivity, and short processes. (Fig. 2a1). Following purification and for third-passage subculture, the purified astrocytes reached over 95% confluence and displayed a characteristic paving stone-like morphology with typically flat and polygonal shapes (Fig. 2a2). Immunofluorescence staining confirmed that nearly all cells were GFAP-positive, with cell purity exceeding 90% (Fig. 2a3), meeting the experimental criteria for subsequent experiments. To simulate in vivo spinal cord injury, a mechanical scratch was applied to the confluent astrocyte monolayers. Within 48 h post-injury, astrocytes at the scratch margin exhibited hypertrophy, extended pseudopodia toward the cell-free zone, and showed marked migration (Fig. 2a4-5). Over the subsequent culture period, the scratch wound gradually closed (Fig. 2b1-d4).

To further characterize astrocyte activation following mechanical insult, we examined the expression of canonical reactive astrocyte markers (GFAP, Vimentin) and chondroitin sulfate proteoglycan (CSPG) components associated with glial scar remodeling (Neurocan, NG2) [35–39]. After scratch injury, GFAP and Vimentin expression levels were robustly upregulated in astrocytes, accompanied by marked cell hypertrophy. The cellular processes became thickened, increased in density, and interwoven to form a dense reticular network (Fig. 2b1-b4). Concurrently, Neurocan and NG2 displayed a diffuse distribution around the scratch wound and cell migration areas (Fig. 2c1-d4). Collectively, the upregulation and spatial redistribution of these signature molecules confirmed the successful establishment of an in vitro GS model. Over time, although the scratch wound widths gradually narrowed, CSPG-related scar markers remained persistently upregulated, indicating that the GS model remains stable and does not resolve spontaneously without external intervention. To confirm that newly generated neurons originated from astrocyte conversion rather than from potential contamination by resident neurons or neural stem/progenitor cells (NSPCs), we performed immunostaining on astrocyte cultures at the same passage used for reprogramming. No expression of the neuronal marker Tuj-1 or the NSPC marker Nestin was detected (Fig. 2e1-f4). Furthermore, all DAPI-positive nuclei were co-localized with GFAP, confirming a highly pure astrocyte population. These results verified the effectiveness of our primary culture and purification protocols in eliminating neuronal and NSPC contaminants. Thus, the Tuj-1-positive neurons observed in later experiments were derived from the direct reprogramming of astrocytes via PBAE nanoparticle-mediated overexpression of the ASCL1 and NGN2.

### Neurogenesis in an in vitro glial scar model following PBAE‑A/N plasmid nanoparticle delivery

**Fig. 3:**
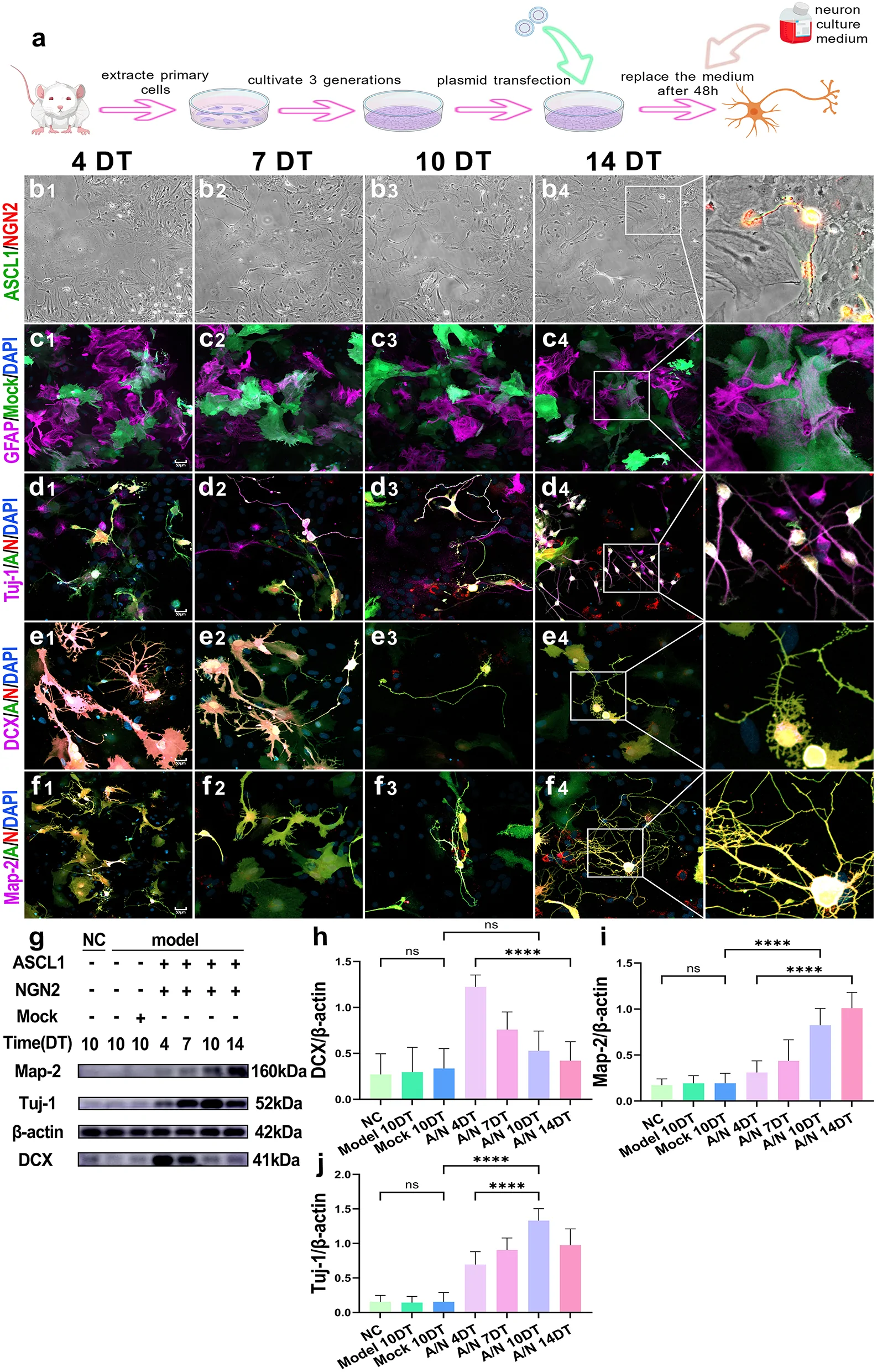
PBAE nanoparticles loaded with ASCL1/NGN2 reprogram astrocytes in the glial scar region. (**a**) Schematic illustration of in vitro astrocyte culture and transfection with ASCL1/NGN2-loaded PBAE nanoparticles. (**b1**-**b4**) Phase-contrast images of astrocytes in the GS region after transfection with ASCL1/NGN2-loaded PBAE nanoparticles (scale bar = 50 μm). (**c1**-**c4**) GFAP immunofluorescence images of cells transfected with mock plasmid (empty vector expressing eGFP). (**d1**-**f4**) Immunofluorescence images of Tuj-1, DCX, and Map-2 in astrocytes co-transfected with ASCL1 (eGFP) and NGN2 (mCherry). scale bar = 50 μm. (**g**-**j**) Western blot and quantitative analysis of Tuj-1, DCX, and Map-2 protein expression following A/N transfection. β-actin served as a loading control. All data are presented as mean ± SD. ^****^P < 0.0001 vs. Mock group, A/N 4 DT vs. A/N 10 DT

To investigate the potential of PBAE-A/N plasmid nanoparticles to reprogram reactive astrocytes into neurons, we delivered the nanoparticles into an established glial scar (GS) model. A detailed schematic of the experimental procedure is presented in Fig. 3a. Following transfection with ASCL1 and NGN2 genes, astrocytes within GS regions underwent prominent morphological transformations. Their originally broad, flattened, and irregular cell bodies contracted and adopted neuron-like shapes. Notably, the cytoplasm near the nucleus gradually concentrated toward the soma, while distal regions of the cell body retracted and elongated into thin, emerging processes. These newly sprouting continued to elongate and branch over time, eventually developing into complex neurite networks (Fig. 3b1-b4). Furthermore, the transfected cells exhibited robust expression of the plasmid-encoded fluorescent reporters (GFP and mCherry).

In contrast, astrocytes in the mock-transfected group, which received vectors expressing only GFP and mCherry without A/N genes, retained their typical flattened morphology with irregular pseudopodia and tight intercellular connections, maintaining a characteristic paving stone-like monolayer consistent with the scratch-only model group (Fig. 2b1–d4). No significant morphological alterations were observed, and these control cells stably expressed the reporter fluorescence throughout the culture period (Fig. 3c1-c4).

To characterize the converted neuron-like cells, we performed systematic immunofluorescence and western blot analysis to detect neuronal markers at days 4, 7, 10, and 14 post-transfection (DT). Tuj-1 expression was rapidly induced following reprogramming initiation, with substantial Tuj-1-positive signals at 4 DT, indicating the progressive acquisition of neuronal fate. Furthermore, Tuj-1 expression levels increased with elongated transduction duration, peaked at 10 DT, and declined at 14 DT (Fig. 3d1-d4). In contrast, doublecortin (DCX) exhibited a transient expression profile at 4 DT but decreased rapidly thereafter and became undetectable by 14 DT (Fig. 3e1-e4). This suggests that the converted cells undergo a brief transition phase prior to maturation. In contrast, Microtubule-Associated Protein 2 (Map-2) expression was barely detectable at 4 DT but increased progressively throughout the observation period, reaching the highest level at 14 DT (Fig. 3f1-f4). By 10 DT, Map-2-positive cells accounted for 86.35% of Tuj-1-positive cells (Supplementary Fig. d-e4). Consistent with immunofluorescence results, western blot analysis (*n* = 3) further confirmed that treatment with PBAE-A/N plasmid nanoparticles robustly upregulated the expression of neuronal markers at the indicated time points. The expression patterns of these markers closely mirrored the immunofluorescence results (Fig. 3j-i). These findings suggest that ASCL1/NGN2-induced neurons do not remain stalled in a precursor state but instead progress toward a more mature phenotype.

### PBAE nanoparticle-mediated reprogramming remodels the glial scar

**Fig. 4:**
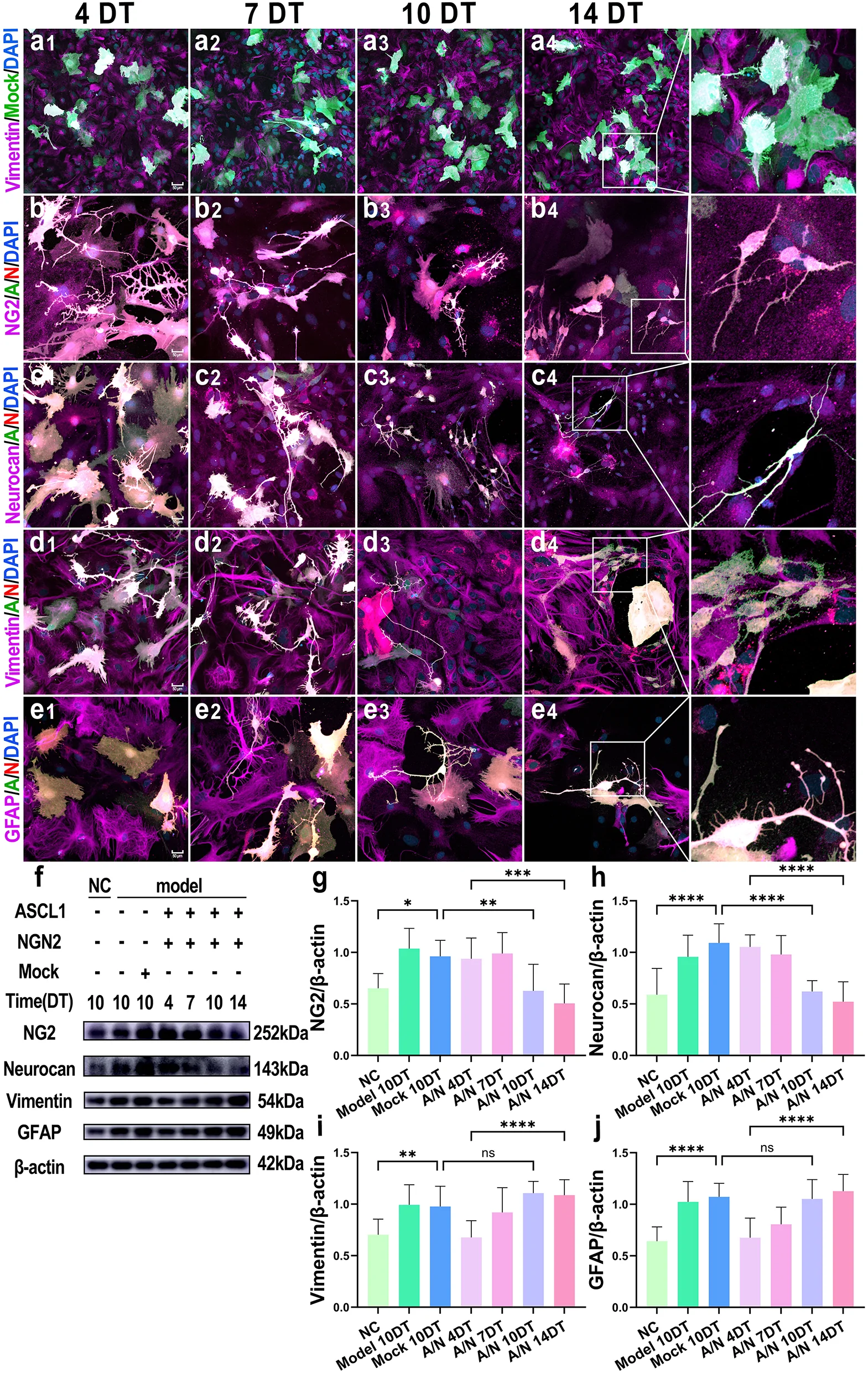
Effects of PBAE nanoparticle-mediated reprogramming on the glial scar dissolution. (**a1**-**a4**) Vimentin immunofluorescence images of cells transfected with a mock plasmid (scale bar = 50 μm). (**b1**-**e4**) Immunofluorescence images of NG2, Neurocan, Vimentin, and GFAP following A/N transfection (scale bar = 50 μm). (**f**-**j**) Western blot analysis and quantification analysis of NG2, Neurocan, Vimentin, and GFAP protein expression following A/N transfection. β-actin served as a loading control. Data are presented as mean ± SD. ^*^P < 0.05, ^**^P < 0.01, ^***^P < 0.001, ^****^P < 0.0001 vs. Mock group

To verify whether PBAE nanoparticle-mediated reprogramming of reactive astrocytes remodels glial scars (GSs), we analyzed the changes in cellular and molecular profiles at multiple time points post-transfection (4, 7, 10, and 14 DT). As shown in Fig. 4a1–a4, astrocytes in the mock group maintained stable morphology and spatial distribution throughout the observation period, displaying a dense, flattened, and confluent monolayer. Immunofluorescence staining revealed high Vimentin expression in eGFP^+^ astrocytes within GSs (as shown in high magnifications in right panels). In contrast, astrocytes in the A/N-mediated group gradually shifted from a flat to a neuron-like morphology. The cell arrangement within GSs became looser due to somatic contraction, reduced proliferation, and acquisition of neuron-like shapes. Notably, NG2 and Neurocan fluorescence intensity decreased with prolonged transduction time in the A/N-mediated group, although a subset of cells retained relatively high expression levels. while a subset of cells retained relatively high expression levels. However, GFAP, NG2, Neurocan, and Vimentin expression persisted in some neuron-like cells at 10DT and 14DT (Fig. 4b1-e4). Western blot analysis (*n* = 3) revealed a phased downregulation of NG2 and Neurocan expression. At early reprogramming stages (4 and 7 DT), NG2 and Neurocan levels remained moderately elevated, similar to those in the control group at 10 DT. By 10 DT, their expression began to decline, reaching the lowest levels at 14 DT (Fig. 4f-h). In contrast, intracellular Vimentin and GFAP expression in the A/N-mediated group increased progressively throughout the experiment, reaching levels comparable to the control group by 10 DT (Fig. 4f, i-j).

### PBAE nanoparticles loaded with ASCL1/NGN2 reprogram astrocytes into diverse neuron subtypes

**Fig. 5:**
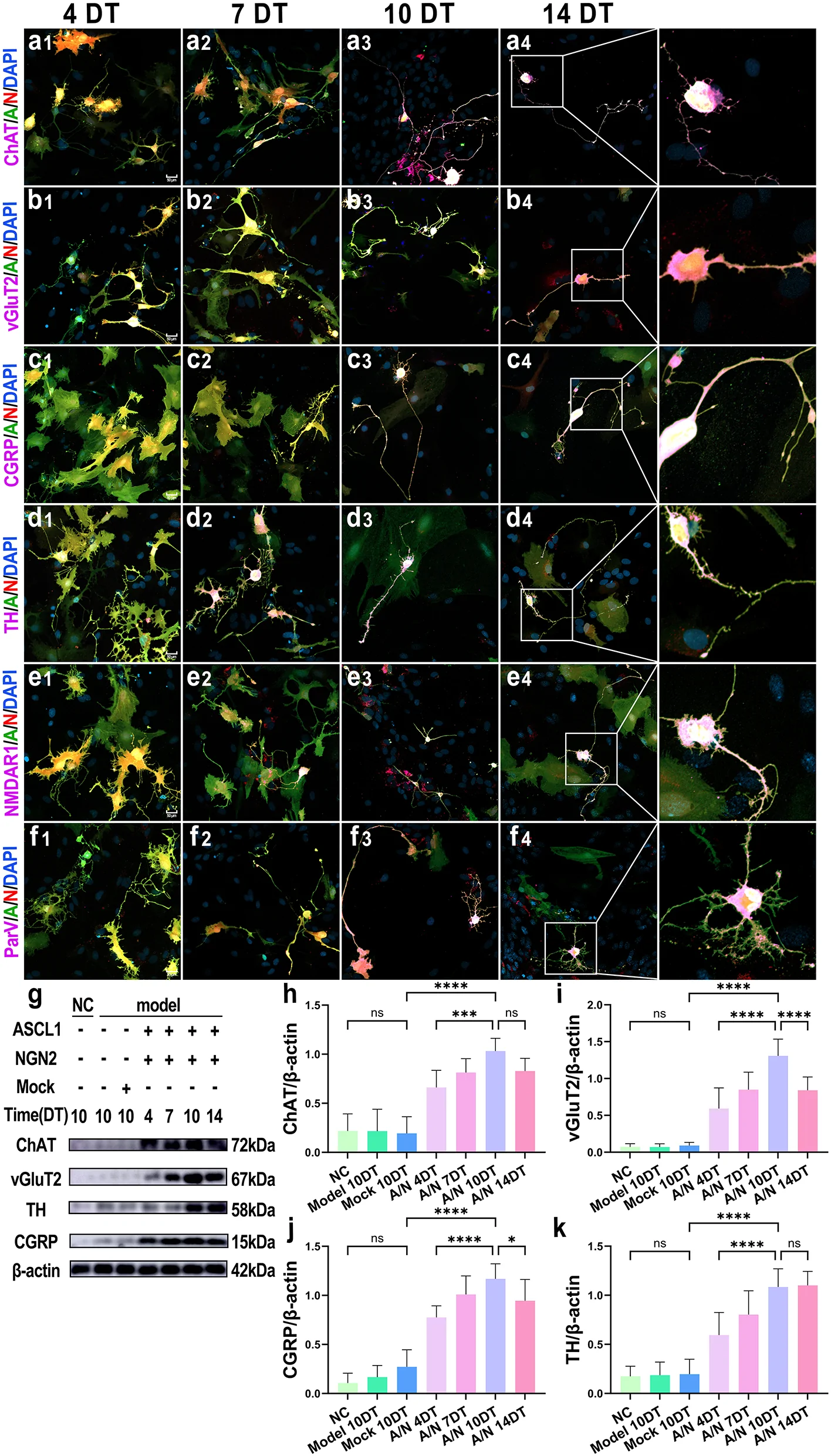
PBAE nanoparticle-mediated reprogramming generates diverse neuronal subtypes. (**a1**-**f4**) Immunofluorescence of CHAT (cholinergic neurons), vGluT2 (glutamatergic neurons), CGRP (peptidergic neurons), tyrosine hydroxylase (TH, dopaminergic neurons), NMDA receptor subunit NR1 (NMDAR1), and parvalbumin (ParV, intermediate neurons) in reprogrammed cells following A/N transfection (scale bar = 50 μm). (**g**-**k**) Western blot analysis and quantification analysis of ChAT, vGluT2, CGRP, and TH protein expression following A/N transfection. β-actin served as a loading control. Data are presented as mean ± SD. ^*^P < 0.05, ^***^P < 0.001, ^****^P < 0.0001 vs. Mock group

To determine the neuronal subtypes generated by PBAE nanoparticle-mediated reprogramming, we performed phenotypic verification via immunofluorescence staining and Western blot analysis. As shown in Fig. 5a1-c4, immunofluorescence showed that PBAE-A/N nanoparticles reprogrammed astrocytes into neuron-like cells expressing neuronal subtype markers ChAT, vGluT2, and CGRP. Western blot analysis (*n* = 3) demonstrated that these markers were first detected at 4 DT, gradually increased over time, peaked at 10 DT, and subsequently declined by 14 DT (Fig. 5h-j). By 10 DT, ChAT-positive and VGLUT2-positive cells accounted for 9.82% and 40.36% of Tuj-1-positive cells, respectively (Figure S2 a1-c4, d-f). We detected tyrosine hydroxylase (TH), a kind of key rate-limiting enzyme for dopaminergic neurons. Western blot analysis (*n* = 3) showed that TH expression also peaked at 10 DT and remained stably elevated through 14 DT (Fig. 5d1-d4, k) Furthermore, immunofluorescence staining revealed that a subset of reprogrammed cells in the GS area were positive for NMDA receptor subunit NR1 (NMDAR1) (Fig. 5e1-e4) and an interneuron marker parvalbumin (ParV) (Fig. 5f1-f4). Collectively, these findings indicate that the reprogrammed neurons comprise multiple subtypes, including cholinergic, glutamatergic, peptidergic, dopaminergic, and intermediate neurons.

### Functional characteristics of neurons reprogrammed from astrocytes

**Fig. 6:**
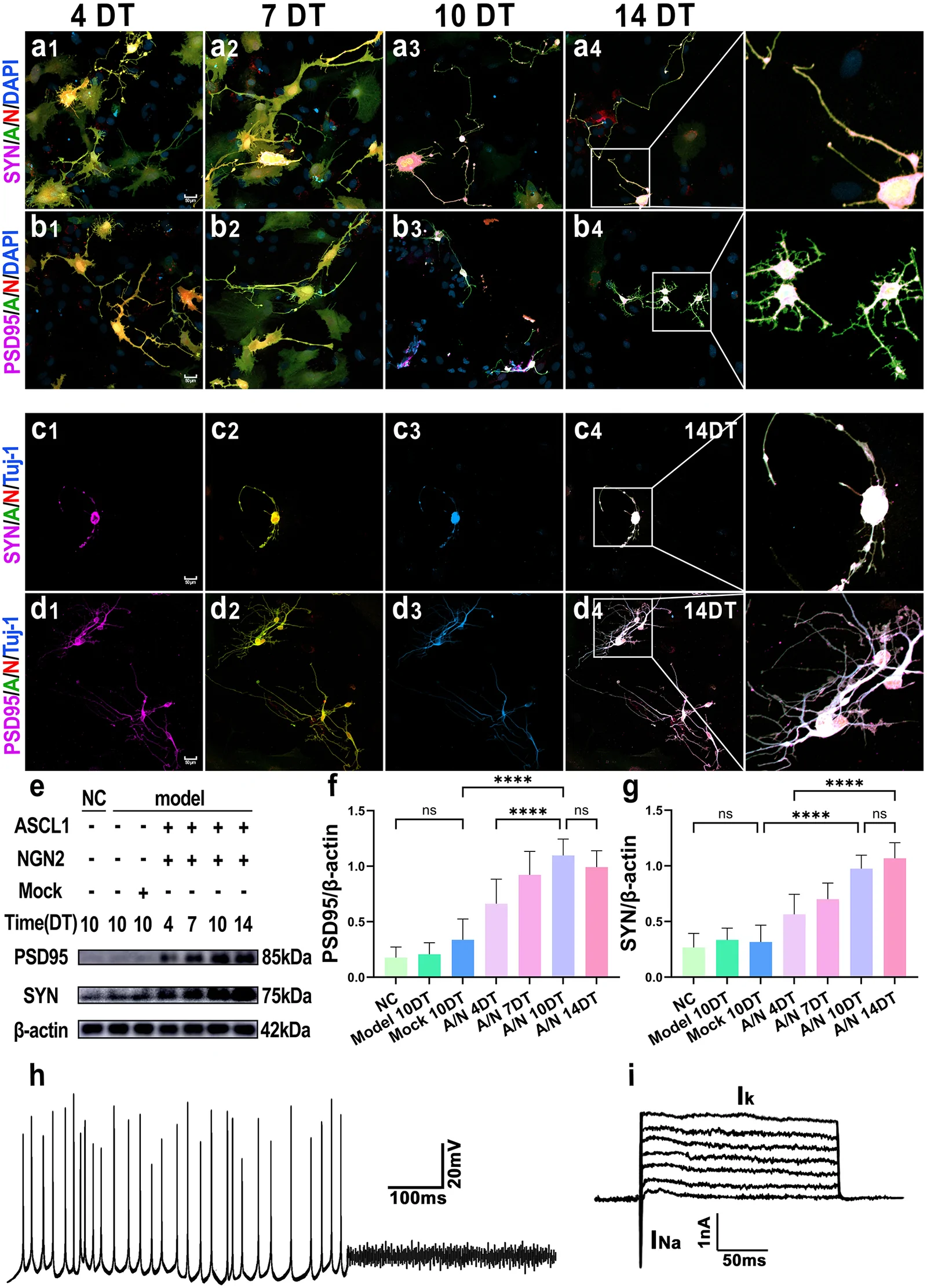
Functional assessment of neurons reprogrammed from astrocytes within GSs. (**a1**-**b4**) Immunofluorescence staining of SYN and PSD95 following PBAE-A/N nanoparticle transfection. scale bar = 50 μm. (**c1**-**d4**) Immunofluorescence co-staining of SYN/Tuj-1 and PSD95/Tuj-1 following PBAE-A/N nanoparticle transfection. scale bar = 50 μm. (**e**-**g**) Western blot images and quantitative analysis of SYN and PSD95 expression post PBAE-A/N nanoparticle transfection. (**h**) Reprogrammed neurons fired irregular and instable spontaneous action potentials (AP) with variable amplitude and frequency. Notably, action potentials were suppressed by TTX. (**i**) Depolarization-evoked sodium and potassium currents recorded at different membrane potentials. Data are presented as mean ± SD. ^****^P < 0.0001 vs. mock group

Although reprogrammed astrocytes exhibited neuronal morphological characteristics and biochemical features, the functional properties of these neuron-like cells remained uncharacterized. To address this, we first examined the expression of synapsin and PSD95, specific markers associated with functional synapses, via immunostaining and western blot. As shown in Fig. 6a1-b4, immunofluorescence staining revealed distinct SYN and PSD95 positive signals in the converted cells at 4 DT, 7 DT, 10 DT and 14 DT following transduction. These signals were localized cell bodies, axons, and dendrites, and the fluorescence intensity elevated progressively over time. Notably, both SYN and PSD95 were co-localized with Tuj-1 and MAP2 (Fig. 6c1-d4). Consistent with the immunofluorescence findings, Western blot analysis (*n* = 3) further revealed SYN expression as early as at 4 DT, peaked at 14 DT (Fig. 6e, g). This successive elevation trend aligned with the expression trend of mature neuronal marker Map-2, indicating gradual maturation of the converted neurons. Strikingly, PSD95 expression exhibited progressive upregulation, peaking at 10DT followed by a slight, non-significant reduction at 14 DT (Fig. 6e, f). The temporal divergence expression suggests distinct regulation for synaptic components during maturation. Next, we examined whether these neuron-like cells possessed intact neuronal electrophysiological properties using whole-cell patch-clamp recording at 14 DT. As shown in Figure 6h-i, the majority of converted neurons (6 out of 8) can fire repetitive action potentials (APs) upon depolarization. Voltage-clamp recordings revealed fast-activating and fast-inactivating inward currents that were blocked by tetrodotoxin (TTX), confirming the presence of functional voltage-gated sodium channels. Of note, the reprogrammed neurons fired irregular spontaneous APs of varying amplitudes, indicative of functional neuronal firing patterns. Additionally, these cells displayed voltage-sensitive inward and outward currents with typical temporal characteristics of sodium and potassium currents (Fig. 6i).

## Discussion

Glial scar (GS) formation and neuronal loss following CNS injury pose significant challenges to neural repair and functional recovery. Although the GS provides initial protective benefits during the acute phase, it subsequently develops into a physical and chemical barrier that severely impedes neural regeneration and axonal regrowth. This inhibition is largely attributed to the secretion of inhibitory extracellular matrix components such as chondroitin sulfate proteoglycans (CSPGs) [40–42]. Therefore, effectively remoding GSs and replenishing lost neurons emerges as a promising therapeutic strategy for neural regeneration and function recovery.

Accumulating evidence has demonstrated that astrocytes can be reprogrammed into neural stem cells (NSCs) or neurons through specific exogenous gene modulation [2, 11, 43], which offers a potential approach to replace lost neurons. Conventional reprogramming strategies often predominantly rely on viral vectors, which are limited by inherent drawbacks including genomic integration risks, high immunogenicity, and constrained cargo capacity [44, 45], hindering clinical translation. In recent years, biodegradable non-viral vectors, particularly poly(β-amino ester) (PBAE) nanoparticles, have attracted considerable interest due to their controllable synthesis, high loading and transfection efficiency, low cytotoxicity, and favorable biocompatibility. In the present study, we further optimized the synthesis protocol to prepare high-molecular-weight PBAE biomaterials with enhanced stability and high loading capacity. By adopting an aqueous assembly system, DNA integrity was maximally preserved, laying a solid foundation for safe and efficient gene delivery. Accordingly, we propose a “remodeling–regeneration integration” therapeutic strategy: optimized PBAE nanoparticles delivering the proneural transcription factors A/N to directly reprogram glial scar astrocytes into functional neuronal subtypes in situ. This approach not only disrupts the physical architecture of GS and reduces inhibitory ECM deposition but also has the potential to replenish lost neurons, providing a novel non-viral therapeutic avenue for CNS injury.

First, the gene delivery vector was optimized by refining the PBAE synthesis protocol. This refinement shortened polymerization time and mitigated polymer degradation and byproduct formation typically caused by prolonged high-temperature heating. The optimized protocol yielded PBAE polymers with markedly elevated molecular weight compared with conventionally synthesized products. These modifications conferred clear functional advantages: longer polymer chains provided greater DNA loading capacity, forming denser and more stable complexes that effectively protect plasmid DNA and substantially improve transfection efficiency in primary astrocytes. Second, a critical methodological improvement was implemented during nanocomplex assembly, replacing the conventional acidic sodium acetate buffer with pure water. Although acidic conditions promote PBAE protonation and DNA condensation, they also compromise DNA integrity, potentially inducing strand breaks or base modification that impair gene expression efficiency and safety [46]. Our acid-free assembly strategy completely avoids acid-induced DNA damage, ensuring the integrity and biological activity of the loaded ASCL1/NGN2 plasmid during delivery. Finally, the inherent biodegradability of PBAE is central to its clinical translation potential. Unlike non-degradable cationic polymers (e.g., polyethyleneimine (PEI)) or integrating viral vectors, PBAE can be hydrolyzed into non-toxic small molecules under physiological conditions after gene delivery [47]. This property enables in vivo metabolic clearance, avoiding chronic inflammation and residual toxicity caused by long-term material accumulation. Moreover, PBAE eliminates the insertional mutagenesis risks associated with viral vectors. Collectively, these improvements in synthesis and formulation yielded a highly efficient, stable, and biocompatible non-viral gene delivery vector with promising clinical prospects. Thus, this system provides reliable technical support for targeted GS reprogramming in the present study and, as a versatile gene delivery platform, holds promise for pioneering nucleic acid-based therapies for other neurological disorders.

To determine the efficacy of PBAE nanoparticles in reprogramming spinal cord glial cells into neurons, we first developed an in vitro SCI model and characterized the phenotypic features of the transformed cells. The results demonstrated that the mechanical scratch wound effectively recapitulated key features of the in vivo glial scar, including astrocyte hypertrophy, proliferation, and progressive expression of characteristic markers (e.g., Vimentin, Neurocan, and NG2), confirming successful model establishment. Following delivery of PBAE-A/N nanoparticles, glial cells within the scar region acquired neuron-like morphology and expressed the early neuronal marker DCX albeit this expression declined rapidly. Concomitantly, Tuj-1 expression initially increased and then declined, while MAP2 levels continuously rose, a trend that was consistent with neuronal development. This dynamic marker expression implicated a gradual phenotypic transition of converted neurons from an immature state toward mature neuronal phenotypes. Collectively, these results indicate that PBAE-A/N nanoparticles effectively induce complete astrocyte-to-neuron reprogramming, driving the progressive maturation of converted neurons. Conversely, immunofluorescence and Western blot analyses revealed significant downregulation of NG2 and Neurocan, key inhibitory extracellular matrix (ECM) components of the GSs, during the reprogramming process. These results demonstrated effective remodeling of the chemical barrier, creating a microenvironment more conducive to neural regeneration and circuit reconstruction. Furthermore, astrocyte-to-neuron reprogramming attenuated the physical barrier of the GS and offered the potential to replenish lost neurons. Together, these findings provide an experimental foundation for the “remodeling–regeneration integrated” therapeutic strategy to promote neural regeneration and function recovery.

Of significance, the neurons generated through this reprogramming process encompassed multiple neuronal subtypes, including cholinergic (ChAT), glutamatergic (vGluT2), sensory peptidergic (CGRP), dopaminergic (TH), and GABAergic (Parvalbumin) neurons. The findings highlight the potential of this approach to address the dynamic nature of SCI-induced neurological deficits, which frequently entail the loss of diverse neuronal populations, including motor, sensory, intermediate, and autonomic subtypes. Existing neuronal replacement strategies predominantly aim to replenish only a certain cell type, thereby restricting the comprehensive reconstruction of intricate neural circuits. In comparison with conventional repair strategies [48–50], the method possesses superior therapeutic potential for neurological injuries, largely owing to its capacity to generate multiple neuronal subtypes. Specifically, cholinergic neurons are responsible for motor functional control; glutamatergic and GABAergic neurons primarily maintain local excitatory-inhibitory balance; and sensory and modulatory neurons may facilitate the restoration of sensory and autonomic functions. Collectively, these results underscore the potential of the PBAE delivery system to confront a fundamental challenge in SCI repair: reconstructing the heterogeneous cellular composition and intricate neural networks within the injured region. Synapse formation and neurophysiological activity are pivotal hallmarks of neuronal functionality. To assess this, we observed expression of Synapsin and PSD-95 in soma and neurites of the converted neuron-like cells. More importantly, these cells exhibited action potential firing and acquired neuron-specific voltage-gated sodium and potassium currents. These findings further support our previous conclusion that PBAE-A/N nanoparticles are critical for efficient reprogramming of astrocytes within GS. Despite irregular spike amplitudes and frequencies, the overall electrophysiological activity of these neurons approximated that of genuine neurons, confirming successful “phenotypic conversion” and “functional acquisition”.

In summary, this study provides proof-of-concept experimental evidence that biodegradable PBAE-A/N nanoparticles can effectively reprogram astrocytes within GSs into distinct neuronal subtypes. Nevertheless, several limitations should be considered: (1) The induced neurons display neurophysiological variability‌, particularly in AP amplitude and frequency. (2) All experiments were performed exclusively in vitro, lacking validation in animal models; thus, the current findings serve as proof-of-concept evidence rather than direct therapeutic validation. (3) The molecular mechanisms underlying A/N-driven reprogramming remain unclear, including how ASCL1 and NGN2 synergistically overcome epigenetic barriers and initiate neuronal programs, as well as the downstream signaling pathways and target gene networks involved. (4) Transfection and reprogramming efficiencies require further improvement to enhance clinical scalability. To address these challenges, future studies will focus on optimizing the physicochemical properties of PBAE (e.g., by incorporating novel side-chain chemical moieties) and identifying superior TF combinations. In addition, systematic in vivo investigations in appropriate SCI animal models are an indispensable prerequisite before any translational inference can be drawn. Notwithstanding these limitations, this study lays a critical theoretical basis for using proneural transcription factors to reprogram autologous glial scar cells into functional neurons. This strategy holds substantial promise for reversing permanent neurological deficits caused by GS and provides a transformative therapeutic approach for comprehensive neural repair.

## Supplementary Information

Below is the link to the electronic supplementary material.

Supplementary Material 1

Supplementary Material 2

## Data Availability

No datasets were generated or analysed during the current study. Data are available from the authors upon request.

## References

[1] Hagen EM. Acute complications of spinal cord injuries. World J Orthop. 2015;6:17–23. 10.5312/wjo.v6.i1.17. PMC4303786 25621207

[2] Pereira A, Diwakar J, Masserdotti G, Beşkardeş S, Simon T, So Y, et al. Direct neuronal reprogramming of mouse astrocytes is associated with multiscale epigenome remodeling and requires Yy1. Nat Neurosci. 2024;27:1260–73. 10.1038/s41593-024-01677-5. PMC11239498 38956165

[3] Jiang B, Sun D, Sun H, Ru X, Liu H, Ge S, et al. Prevalence, Incidence, and External Causes of Traumatic Spinal Cord Injury in China: A Nationally Representative Cross-Sectional Survey. Front Neurol. 2022;12:784647. 10.3389/fneur.2021.784647. PMC8811043 35126291

[4] Chen C, Qiao X, Liu W, Fekete C, Reinhardt JD. Epidemiology of spinal cord injury in China: A systematic review of the chinese and english literature. Spinal Cord Nat Publishing Group. 2022;60:1050–61. 10.1038/s41393-022-00826-6. 35778501

[5] Fleming JC, Norenberg MD, Ramsay DA, Dekaban GA, Marcillo AE, Saenz AD, et al. The cellular inflammatory response in human spinal cords after injury. Brain. 2006;129:3249–69. 10.1093/brain/awl296. 17071951

[6] Q RF, Jj SPX. L, Y T. Phagocytosis of microglia in the central nervous system diseases. Molecular neurobiology [Internet]. Mol Neurobiol; 2014 [cited 2024 Sept 23];49. 10.1007/s12035-013-8620-6 PMC4012154 24395130

[7] Yang T, Dai Y, Chen G, Cui S. Dissecting the Dual Role of the Glial Scar and Scar-Forming Astrocytes in Spinal Cord Injury. Front Cell Neurosci. 2020;14:78. 10.3389/fncel.2020.00078. PMC7147295 32317938

[8] Masserdotti G, Gillotin S, Sutor B, Drechsel D, Irmler M, Jørgensen HF, et al. Transcriptional mechanisms of proneural factors and REST in regulating neuronal reprogramming of astrocytes. Cell Stem Cell. 2015;17:74–88. 10.1016/j.stem.2015.05.014. PMC4509553 26119235

[9] Rao Z, Wang R, Li S, Shi Y, Mo L, Han S, et al. Molecular mechanisms underlying Ascl1-mediated astrocyte-to-neuron conversion. Stem Cell Rep. 2021;16:534–47. 10.1016/j.stemcr.2021.01.006. PMC7940254 33577795

[10] Guo Z, Zhang L, Wu Z, Chen Y, Wang F, Chen G. In vivo direct reprogramming of reactive glial cells into functional neurons after brain injury and in an alzheimer’s disease model. Cell Stem Cell. 2014;14:188–202. 10.1016/j.stem.2013.12.001. PMC3967760 24360883

[11] Ma Y, Xie H, Du X, Wang L, Jin X, Zhang Q, et al. In vivo chemical reprogramming of astrocytes into neurons. Cell Discovery. 2021;7:12. 10.1038/s41421-021-00243-8. PMC7921425 33649311

[12] Tan Z, Qin S, Liu H, Huang X, Pu Y, He C, et al. Small molecules reprogram reactive astrocytes into neuronal cells in the injured adult spinal cord. J Adv Res. 2023;59:111–27. 10.1016/j.jare.2023.06.013. PMC11081968 37380102

[13] Yin J-C, Zhang L, Ma N-X, Wang Y, Lee G, Hou X-Y, et al. Chemical conversion of human fetal astrocytes into neurons through modulation of multiple signaling pathways. Stem Cell Rep. 2019;12:488–501. 10.1016/j.stemcr.2019.01.003. PMC6409415 30745031

[14] Di Rivetti P, Romanov RA, Spigolon G, Masini D, Martín-Montañez E, Toledo EM, et al. Induction of functional dopamine neurons from human astrocytes in vitro and mouse astrocytes in a parkinson’s disease model. Nat Biotechnol. 2017;35:444–52. 10.1038/nbt.3835. 28398344

[15] Tai W, Wu W, Wang L-L, Ni H, Chen C, Yang J, et al. In vivo reprogramming of NG2 glia enables adult neurogenesis and functional recovery following spinal cord injury. Cell Stem Cell. 2021;28:923–e9374. 10.1016/j.stem.2021.02.009. PMC8106641 33675690

[16] Li W, Su D, Li X, Lu K, Huang Q, Zheng J, et al. Identification of the core regulatory program driving NEUROD1-induced neuronal reprogramming. Cell Rep. 2025;44. Available from: 10.1016/j.celrep.2025.115523. 40173039

[17] Chen Y-C, Ma N-X, Pei Z-F, Wu Z, Do-Monte FH, Keefe S, et al. A NeuroD1 AAV-based gene therapy for functional brain repair after ischemic injury through in vivo astrocyte-to-neuron conversion. Mol Ther. 2020;28:217–34. 10.1016/j.ymthe.2019.09.003. PMC6952185 31551137

[18] Wapinski OL, Lee QY, Chen AC, Li R, Corces MR, Ang CE, et al. Rapid Chromatin Switch in the Direct Reprogramming of Fibroblasts to Neurons. Cell Rep. 2017;20:3236–47. 10.1016/j.celrep.2017.09.011. PMC5646379 28954238

[19] Chanda S, Ang CE, Davila J, Pak C, Mall M, Lee QY, et al. Generation of induced neuronal cells by the single reprogramming factor ASCL1. Stem Cell Rep. 2014;3:282–96. 10.1016/j.stemcr.2014.05.020. PMC4176533 25254342

[20] Parras CM, Schuurmans C, Scardigli R, Kim J, Anderson DJ, Guillemot F. Divergent functions of the proneural genes Mash1 and Ngn2 in the specification of neuronal subtype identity. Genes Dev. 2002;16:324–38. 10.1101/gad.940902. PMC155336 11825874

[21] Kenjo E, Hozumi H, Makita Y, Iwabuchi KA, Fujimoto N, Matsumoto S, et al. Low immunogenicity of LNP allows repeated administrations of CRISPR-Cas9 mRNA into skeletal muscle in mice. Nat Commun Nat Publishing Group. 2021;12:7101. 10.1038/s41467-021-26714-w. PMC8654819 34880218

[22] Nidetz NF, McGee MC, Tse LV, Li C, Cong L, Li Y, et al. Adeno-associated viral vector-mediated immune responses: understanding barriers to gene delivery. 2020. Available from: 10.1016/j.pharmthera.2019.107453 PMC6980784 31836454

[23] Shirley JL, de Jong YP, Terhorst C, Herzog RW. Immune Responses to Viral Gene Therapy Vectors. Mol Ther. 2020;28:709–22. 10.1016/j.ymthe.2020.01.001. PMC7054714 31968213

[24] Cordeiro RA, Serra A, Coelho JFJ, Faneca H. Poly(β-amino ester)-based gene delivery systems: From discovery to therapeutic applications. J Control Release. 2019;310:155–87. 10.1016/j.jconrel.2019.08.024. 31454533

[25] Iyer AK, Singh A, Ganta S, Amiji MM. Role of integrated cancer nanomedicine in overcoming drug resistance. Adv Drug Deliv Rev. 2013;65:1784–802. 10.1016/j.addr.2013.07.012. 23880506

[26] Y L, Y L, D K, L S. Poly(β-amino esters): synthesis, formulations, and their biomedical applications. Adv Healthc Mater [Internet]. 2019 [cited 2024 Oct 13];8. Available from: 10.1002/adhm.201801359. 30549448

[27] Yang H, Cheng X-P, Li J-W, Yao Q, Ju G. De-differentiation response of cultured astrocytes to injury induced by scratch or conditioned culture medium of scratch-insulted astrocytes. Cell Mol Neurobiol. 2009;29:455–73. 10.1007/s10571-008-9337-3. PMC11505826 19130217

[28] Feng G-D, He B-R, Lu F, Liu L-H, Zhang L, Chen B, et al. Fibroblast growth factor 4 is required but not sufficient for the astrocyte dedifferentiation. Mol Neurobiol. 2014;50:997–1012. 10.1007/s12035-014-8649-1. 24510312

[29] Yang H, Liu Y, Hai Y, Guo Y, Yang S, Li Z, et al. Efficient conversion of spermatogonial stem cells to phenotypic and functional dopaminergic neurons via the PI3K/akt and P21/Smurf2/Nolz1 pathway. Mol Neurobiol. 2015;52:1654–69. 10.1007/s12035-014-8966-4. 25373443

[30] Son EY, Ichida JK, Wainger BJ, Toma JS, Rafuse VF, Woolf CJ, et al. Conversion of mouse and human fibroblasts into functional spinal motor neurons. Cell Stem Cell. 2011;9:205–18. 10.1016/j.stem.2011.07.014. PMC3188987 21852222

[31] Mangraviti A, Tzeng SY, Kozielski KL, Wang Y, Jin Y, Gullotti D, et al. Polymeric Nanoparticles for Nonviral Gene Therapy Extend Brain Tumor Survival in Vivo. ACS Nano. 2015;9:1236–49. 10.1021/nn504905q. PMC4342728 25643235

[32] Sunshine JC, Akanda MI, Li D, Kozielski KL, Green JJ. Effects of base polymer hydrophobicity and end group modification on polymeric gene delivery. Biomacromolecules. 2011;12:3592–600. 10.1021/bm200807s. PMC3959121 21888340

[33] Tzeng SY, Guerrero-Cázares H, Martinez EE, Sunshine JC, Quiñones-Hinojosa A, Green JJ. Non-viral gene delivery nanoparticles based on poly(β-amino esters) for treatment of glioblastoma. Biomaterials. 2011;32:5402–10. 10.1016/j.biomaterials.2011.04.016. PMC3118545 21536325

[34] Niu G, Jin Z, Zhang C, He D, Gao X, Zou C, et al. An effective vaginal gel to deliver CRISPR/Cas9 system encapsulated in poly (β-amino ester) nanoparticles for vaginal gene therapy. EBioMedicine. 2020;58:102897. 10.1016/j.ebiom.2020.102897. PMC7387785 32711250

[35] Yang X. Chondroitin sulfate proteoglycans: key modulators of neuronal plasticity, long-term memory, neurodegenerative, and psychiatric disorders. Rev Neurosci. 2020;31:555–68. 10.1515/revneuro-2019-0117. 32126020

[36] McKeon RJ, Jurynec MJ, Buck CR. The chondroitin sulfate proteoglycans neurocan and phosphacan are expressed by reactive astrocytes in the chronic CNS glial scar. J Neurosci. 1999;19:10778–88. 10.1523/JNEUROSCI.19-24-10778.1999. PMC6784959 10594061

[37] Tan AM, Zhang W, Levine JM. NG2: a component of the glial scar that inhibits axon growth. J Anat. 2005;207:717–25. 10.1111/j.1469-7580.2005.00452.x. PMC1571583 16367799

[38] Sandvig A, Berry M, Barrett LB, Butt A, Logan A. Myelin-, reactive glia-, and scar-derived CNS axon growth inhibitors: expression, receptor signaling, and correlation with axon regeneration. Glia. 2004;46:225–51. 10.1002/glia.10315. 15048847

[39] Buss A, Pech K, Kakulas BA, Martin D, Schoenen J, Noth J, et al. NG2 and phosphacan are present in the astroglial scar after human traumatic spinal cord injury. BMC Neurol. 2009;9:32. 10.1186/1471-2377-9-32. PMC2725028 19604403

[40] Tran AP, Warren PM, Silver J. The Biology of Regeneration Failure and Success After Spinal Cord Injury. Physiol Rev. 2018;98:881–917. 10.1152/physrev.00017.2017. PMC5966716 29513146

[41] Sk KV, M G. M, G M, D S. Spinal cord injury: pathophysiology, treatment strategies, associated challenges, and future implications. Cell and tissue research [Internet]. Cell Tissue Res; 2019 [cited 2024 Sept 23];377. 10.1007/s00441-019-03039-1 31065801

[42] K A-K SDHK, S K-A. JS. LAR and PTPσ receptors are negative regulators of oligodendrogenesis and oligodendrocyte integrity in spinal cord injury. Glia [Internet] Glia. 2019. 10.1002/glia.23533. [cited 2024 Sept 23];67. 30394599

[43] Wei J, Wang M, Li S, Han R, Xu W, Zhao A, et al. Reprogramming of astrocytes and glioma cells into neurons for central nervous system repair and glioblastoma therapy. Biomed Pharmacother. 2024;176:116806. 10.1016/j.biopha.2024.116806. 38796971

[44] Pavlou M, Probst M, Kaplan L, Filippova E, Prieve AR, Rieke F, et al. AAV-mediated expression of proneural factors stimulates neurogenesis from adult müller glia in vivo. EMBO Mol Med. 2025;17:722–46. 10.1038/s44321-025-00209-3. PMC11982270 40050705

[45] Zhang L, Chen X, Wang X, Zhou Y, Fang Y, Gu X, et al. AAV-mediated gene cocktails enhance supporting cell reprogramming and hair cell regeneration. Adv Sci. 2024;11:2304551. 10.1002/advs.202304551. PMC11304307 38810137

[46] Yoshimura H, Ikeda Y, Yoshimoto M, Tamaki S, Hanada K, Kusano T, et al. Structural and functional analysis of a polyoma-related mammalian plasmid (L factor): the enhancer activity and plasmid establishment. Nucleic Acids Res. 1991;19:3633–9. 10.1093/nar/19.13.3633. PMC328391 1649455

[47] Wei J, Zhu L, Lu Q, Li G, Zhou Y, Yang Y, et al. Recent progress and applications of poly(beta amino esters)-based biomaterials. J Control Release: Off J Control Release Soc. 2023;354:337–53. 10.1016/j.jconrel.2023.01.002. 36623697

[48] Tian T, Zhang S, Yang M. Recent progress and challenges in the treatment of spinal cord injury. Protein Cell. 2023;14:635–52. 10.1093/procel/pwad003. PMC10501188 36856750

[49] Kong G, Liu J, Wang J, Yu X, Li C, Deng M, et al. Engineered extracellular vesicles modified by angiopep-2 peptide promote targeted repair of spinal cord injury and brain inflammation. ACS Nano. 2025;19:4582–600. 10.1021/acsnano.4c14675. PMC11803916 39853366

[50] Liu W, Ma Z, Li J, Kang X. Mesenchymal stem cell-derived exosomes: therapeutic opportunities and challenges for spinal cord injury. Stem Cell Res Ther. 2021;12:102. 10.1186/s13287-021-02153-8. PMC7860030 33536064

